# A review of eye tracking for understanding and improving diagnostic interpretation

**DOI:** 10.1186/s41235-019-0159-2

**Published:** 2019-02-22

**Authors:** Tad T. Brunyé, Trafton Drew, Donald L. Weaver, Joann G. Elmore

**Affiliations:** 1 Center for Applied Brain and Cognitive Sciences, Tufts University, 200 Boston Ave., Suite 3000, Medford, MA 02155 USA; 2 Department of Psychology, University of Utah, 380 1530 E, Salt Lake City, UT 84112 USA; 3 Department of Pathology and University of Vermont Cancer Center, University of Vermont, 111 Colchester Ave., Burlington, VT 05401 USA; 4 Department of Medicine, David Geffen School of Medicine at UCLA, University of California at Los Angeles, 10833 Le Conte Ave., Los Angeles, CA 90095 USA

**Keywords:** Eye tracking, Medical informatics, Visual perception, Visual search, Medical decision-making

## Abstract

Inspecting digital imaging for primary diagnosis introduces perceptual and cognitive demands for physicians tasked with interpreting visual medical information and arriving at appropriate diagnoses and treatment decisions. The process of medical interpretation and diagnosis involves a complex interplay between visual perception and multiple cognitive processes, including memory retrieval, problem-solving, and decision-making. Eye-tracking technologies are becoming increasingly available in the consumer and research markets and provide novel opportunities to learn more about the interpretive process, including differences between novices and experts, how heuristics and biases shape visual perception and decision-making, and the mechanisms underlying misinterpretation and misdiagnosis. The present review provides an overview of eye-tracking technology, the perceptual and cognitive processes involved in medical interpretation, how eye tracking has been employed to understand medical interpretation and promote medical education and training, and some of the promises and challenges for future applications of this technology.

## Significance

During patient examinations, image interpretation, and surgical procedures, physicians are constantly accumulating multisensory evidence when inspecting information and ultimately arriving at a diagnostic interpretation. Eye-tracking research has shed light on the dynamics of this interpretive process, including qualitative and quantitative differences that help distinguish and possibly predict successes and errors. This progress affords novel insights into how the interpretive process might be improved and sustained during education, training, and clinical practice. The present review details some of this research and emphasizes future directions that may prove fruitful for scientists, educators, and clinical practitioners interested in accelerating the transition from novice to expert, monitoring and maintaining competencies, developing algorithms to automate error detection and classification, and informing tractable remediation strategies to train the next generation of diagnosticians.

## Introduction

Decades of research have demonstrated the involvement of diverse perceptual and cognitive processes during medical image interpretation and diagnosis (Bordage, 1999; Elstein, Shulman, & Sprafka, 1978; Gilhooly, 1990; Kundel & La Follette, 1972; Patel, Arocha, & Zhang, 2005). Broadly speaking, these include visual search and pattern matching, hypothesis generation and testing, and reasoning and problem-solving. As with many more general cognitive tasks, these processes interact dynamically over time via feed-forward and feed-back mechanisms to guide interpretation and decision-making (Brehmer, 1992; Newell, Lagnado, & Shanks, 2015). The reliable involvement of these processes has made them of interest as targets for both clinical research and the design of educational interventions to improve diagnostic decision-making (Crowley, Naus, Stewart, & Friedman, 2003; Custers, 2015; Nabil et al., 2013). Methodologies to investigate mental processes during interpretation and diagnosis have included think-aloud protocols (Lundgrén-Laine & Salanterä, 2010), knowledge and memory probes (Gilhooly, 1990; Patel & Groen, 1986), practical exercises (Bligh, Prideaux, & Parsell, 2001; Harden, Sowden, & Dunn, 1984), and tracking physicians’ interface navigation behavior while they inspect visual images (e.g., radiographs, histology slides) (Mercan et al., 2016; Mercan, Shapiro, Brunyé, Weaver, & Elmore, 2017).

Medical researchers have increasingly turned to eye-tracking technology to provide more detailed qualitative and quantitative assessments of how and where the eyes move during interpretation, extending research from other high-stakes domains such as air-traffic control (Martin, Cegarra, & Averty, 2011) and airport luggage screening (McCarley & Carruth, 2004; McCarley, Kramer, Wickens, Vidoni, & Boot, 2004). Studies in the medical domain have provided more nuanced understandings of visual interpretation and diagnostic decision-making in diverse medical specialties including radiology, pathology, pediatrics, surgery, and emergency medicine (Al-Moteri, Symmons, Plummer, & Cooper, 2017; Blondon & Lovis, 2015; van der Gijp et al., 2017). Eye tracking has the potential to revolutionize clinical practice and medical education, with far-reaching implications for the development of automated competency assessments (Bond et al., 2014; Krupinski, Graham, & Weinstein, 2013; Richstone et al., 2010; Tien et al., 2014), advanced clinical tutorials (e.g., watching an expert’s eye movements over an image; (Khan et al., 2012; O’Meara et al., 2015)), biologically inspired artificial intelligence to enhance computer-aided diagnosis (Buettner, 2013; Young & Stark, 1963), and the automated detection and mitigation of emergent interpretive errors during the diagnostic process (Ratwani & Trafton, 2011; Tourassi, Mazurowski, Harrawood, & Krupinski, 2010; Voisin, Pinto, Morin-Ducote, Hudson, & Tourassi, 2013).

## Eye tracking: technologies and metrics

**Fig. 1:**
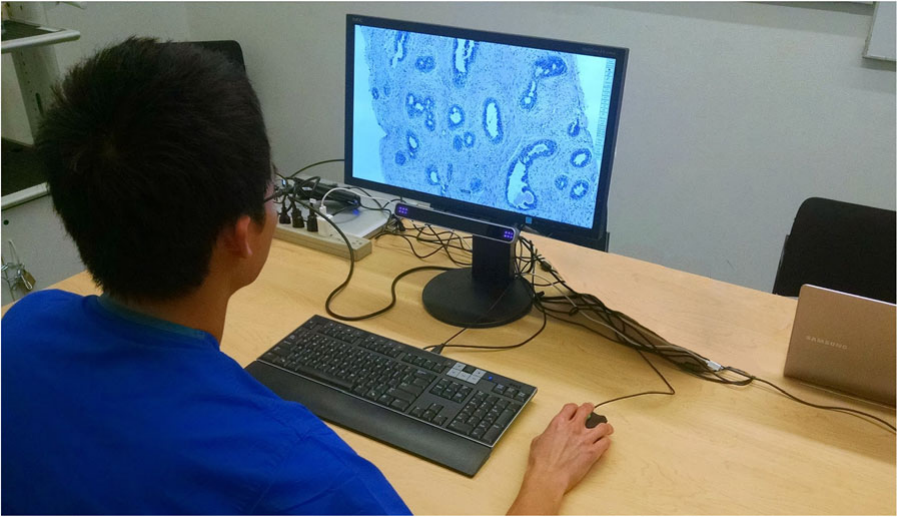
A remote eye-tracking system (SensoMotoric Instruments’ Remote Eye-tracking Device – mobile; SMI REDm) mounted to the bottom of a computer monitor. In this study, a participating pathologist is inspecting a digital breast biopsy (Brunyé, Mercan, et al., 2017)

Modern eye tracking involves an array of infrared or near-infrared light sources and cameras that track the gaze behavior of one (monocular) or both (binocular) eyes (Holmqvist et al., 2011). In most modern systems, an array of non-visible light sources illuminate the eye and produce a corneal reflection (the first Purkinje image); the eye tracker monitors the relationship between this reflection and the center of the pupil to compute vectors that relate eye position to locations in the perceived world (Hansen & Ji, 2010). As the eyes move, the computed point of regard in space also moves. Eye trackers are available in several hardware configurations, including systems with a chin rest for head stabilization, remote systems that can accommodate a limited extent of head movements, and newer mobile eye-wear based systems. Each of these form factors has relative advantages and disadvantages for spatial accuracy (i.e., tracking precision), tracking speed, mobility, portability, and cost (Funke et al., 2016; Holmqvist, Nyström, & Mulvey, 2012). Figure 1 depicts a relatively mobile and contact-free eye-tracking system manufactured by SensoMotoric Instruments (SMI; Berlin, Germany), the Remote Eye-tracking Device – mobile (REDm).

Eye trackers provide several measures of visual behavior that are relevant for understanding the interpretive process; these are categorically referred to as movement measures, position measures, numerosity measures, and latency measures (Holmqvist et al., 2011). Before describing these, it is important to realize that the eye is constantly moving between points of fixation. Fixations are momentary pauses of eye gaze at a spatial location for a minimum amount of time (e.g., > 99 ms), and the movements between successive fixations are called saccades (Liversedge & Findlay, 2000). Movement measures quantify the patterns of eye movements through space during saccades, including the distance between successive saccades (degrees of saccade amplitude) and the speed of saccades (typically average or peak velocity). Position measures quantify the location of the gaze in Cartesian coordinate space, such as the coordinate space of a computer monitor, or a real-world scene captured through a forward-view camera. Numerosity measures quantify the frequency with which the eyes fixate and saccade while perceiving a scene, such as how many fixations and saccades have occurred during a given time, and how those counts might vary as a function of position (and the visual information available at different positions). Finally, latency measures allow for an assessment of the temporal dynamics of fixations and saccades, including first and subsequent fixation durations and saccade duration. Table 1 provides an overview of commonly used eye-tracking measures, and current theoretical perspectives on their relationships to perceptual and cognitive processing.

**Table 1:** A taxonomy relating commonly used eye-tracking metrics and their respective units to perceptual and cognitive processes of interest to researchers

Measure	Units	Description
Fixation count	Frequency count	The number of times the eye fixates in a particular region of interest, related to at least: the salience of the area, the informational value of the area, how much information is available in a single fixation, or the processing difficulty of the information (Findlay & Gilchrist, 2008; Henderson & Hollingworth, 1998; Henderson, Malcolm, & Schandl, 2009)
Regressive fixation count	Frequency count	Re-fixating a previously fixated region, to resolve ambiguity or other processing difficulties (Spivey & Tanenhaus, 1998; Underwood & Radach, 1998)
Fixation duration	Milliseconds	How long the eye fixates on a region prior to a saccade, related to the difficulty in processing the information in that region, the value of information available in that region, the time needed to plan the next saccade, and the predicted value of information available following the next saccade (Findlay & Gilchrist, 2008; Rayner, 1998; Sumner, 2011)
Amplitude	Degrees	The magnitude of a saccade, influenced by how much information can be processed in the area of a single fixation, and the distance to the next planned fixation target (Rayner, 1998)
Saccade peak velocity	Degrees/second	The maximum speed achieved within a saccade, related to physiological arousal, mental workload, or the predicted value of information available at the subsequent fixation (Di Stasi, Catena, Cañas, Macknik, & Martinez-Conde, 2013; Montagnini & Chelazzi, 2005; Xu-Wilson et al., 2009)
Blink rate or inter-blink interval	Frequency count/time or milliseconds	The number of eye blinks detected by an eye tracker’s algorithms, inversely related to physiological arousal, wakefulness, processing difficulty, motivation, and mental workload (Holmqvist et al., 2011; Siegle, Ichikawa, & Steinhauer, 2008)
Blink amplitude and blink duration	Milliseconds	The extent and duration of an eye blink (temporary closure) event, inversely related to physiological arousal, wakefulness, processing difficulty, motivation, and mental workload (Holmqvist et al., 2011; Ingre, Åkerstedt, Peters, Anund, & Kecklund, 2006).
Phasic pupil diameter	Millimeter diameter	Rapid and dramatic pupil diameter changes related to processing task- and goal-relevant information, and exploiting that information to perform a task (Beatty, 1982; Laeng, Sirois, & Gredeback, 2012)
Tonic pupil diameter	Millimeter diameter	Sustained pupil diameter changes that establish a new baseline diameter from which phasic responses deviate, related to sustained cognitive processing, task difficulty, cognitive effort, arousal, and vigilance (Laeng et al., 2012; Siegle et al., 2008).

### Eye tracking in medical interpretation

Some of the earliest research using eye tracking during medical image interpretation was done during x-ray film inspection (Kundel & Nodine, 1978). In this task, radiologists search chest x-ray films for evidence of lung nodules; Kundel and Nodine were interested in whether radiologists were making errors of visual search versus errors of recognition and/or decision-making. A search error would be evidenced by a failure to fixate on a nodule, and a recognition or decision error would occur when a fixation on a nodule is not followed by a successful identification and diagnosis. To further differentiate errors of recognition versus decision-making, Kundel and Nodine distinguished trials where the radiologist fixated within 2.8° of a nodule for greater than or less than 600 ms. If the fixation occurred for less than 600 ms this was considered a recognition error, and if greater than 600 ms it was considered a decision error. The former was considered a failure to disembed the nodule from the background noise (despite fixating on it), and the latter was considered a successful recognition of a nodule without appropriately mapping it to diagnostic criteria. Their results demonstrated that about 30% of all errors were due to a failed search. About 25% of errors were due to a recognition failure, and the remaining 45% of errors were due to decision failure. Thus, interpretive errors were primarily driven by failures of recognition and decision-making, rather than failures of search (Kundel & Nodine, 1978). In other words, radiologists would fixate upon and process the critical visual information in a scene but fail to successfully map that information to known schemas and/or candidate diagnoses. A follow-up study confirmed that fixations over 300 ms did not improve recognition, but did improve decision accuracy; furthermore, fixations within 2° of the nodule were associated with higher recognition accuracy (Carmody, Nodine, & Kundel, 1980). These early studies suggest that eye tracking can be a valuable tool for helping dissociate putative sources of error during medical image interpretation (i.e., search, recognition, and decision-making), given that high-resolution foveal vision appears to be critical for diagnostic interpretation.

Over the past four decades since this original research, eye tracking has been expanded to understanding diagnostic interpretation in several medical specializations, including radiology, breast pathology, general surgery, neurology, emergency medicine, anesthesiology, ophthalmology, and cardiology (Balslev et al., 2012; Berbaum et al., 2001; Brunyé et al., 2014; Giovinco et al., 2015; Henneman et al., 2008; Jungk, Thull, Hoeft, & Rau, 2000; Krupinski et al., 2006; Kundel, Nodine, Krupinski, & Mello-Thoms, 2008; Matsumoto et al., 2011; O’Neill et al., 2011; Sibbald, de Bruin, Yu, & van Merrienboer, 2015; Wood, Batt, Appelboam, Harris, & Wilson, 2014). In general, these eye-tracking studies have found evidence of reliable distinctions between three types of error-making in diagnostic interpretation: search errors, recognition errors, and decision errors. Each of these error types carries implications for diagnostic accuracy and, ultimately, patient quality of life and well-being. We review each of these in turn, below.

#### Search errors

A search error occurs when the eyes fail to fixate a critical region of a visual scene, rendering a feature undetected; these have also been labeled as scanning errors because the critical feature was not in the scan path (Cain, Adamo, & Mitroff, 2013). For example, a radiologist failing to fixate a lung nodule (Manning, Ethell, Donovan, & Crawford, 2006), a pathologist failing to fixate large nucleoli in pleomorphic cells (Brunyé, Mercan, Weaver, & Elmore, 2017), or a neuro-radiologist failing to fixate a cerebral infarction (Matsumoto et al., 2011). Theoretically, if the diagnostician has not fixated a diagnostically relevant region of a medical image then successful search has not occurred, and without it, recognition and decision-making are not possible.

Several perceptual and cognitive mechanisms have been proposed to account for why search errors occur, including low target prevalence, satisfaction of search, distraction, and resource depletion. *Low target prevalence* refers to a situation when a diagnostic feature is especially rare. For example, a malignant tumor appearing in a screening mammography examination has a very low prevalence rate at or below 1% of all cases reviewed (Gur et al., 2004). Low prevalence is associated with higher rates of search failure; previous research has shown that when target prevalence was decreased from 50 to 1%, detection rates fell from approximately 93 to 70%, respectively (Wolfe, Horowitz, & Kenner, 2005). Although much of the research on the low prevalence effect has focused on basic findings with naïve subjects, research has also shown that low prevalence also influences diagnostic accuracy in a medical setting (Egglin & Feinstein, 1996; Evans, Birdwell, & Wolfe, 2013). Most notably, Evans and colleagues compared performance under typical laboratory conditions, where target prevalence is high (50% of cases), and when the same cases were inserted into regular workflow, where target prevalence is low (< 1% of cases) they found that false-negative rates were substantially elevated at low target prevalence (Evans et al., 2013). As a diagnostician searches a medical image, they must make a decision of when to terminate a search (Chun & Wolfe, 1996; Hong, 2005). In the case of low target prevalence, search termination is more likely to occur prior to detecting a target (Wolfe & Van Wert, 2010).

How exactly a search termination decision emerges during a diagnostician’s visual search process is unknown, though it is likely that there are multiple smaller decisions occurring during the search process: as the diagnostician detects individual targets in the medical image, they must decide whether it is the most diagnostically valuable target (and thus terminate search), or whether they believe there is a rare but more valuable target that might be found with continued search (Rich et al., 2008). The risk is that after finding a single target a diagnostician may terminate search prematurely and fail to detect a target with higher value for a correct diagnosis. This phenomenon was originally coined *satisfaction of search*, when radiologists would become satisfied with their interpretation of a medical image after identifying one lesion, at the expense of identifying a second more important lesion (Berbaum et al., 1990; Smith, 1967). These sorts of errors may be a consequence of Bayesian reasoning based on prior experience: the diagnostician may not deem additional search time justifiable for a target that is exceedingly unlikely to be found (Cain, Vul, Clark, & Mitroff, 2012). More recently, Berbaum and colleagues demonstrated that satisfaction of search alone may not adequately describe the search process (Berbaum et al., 2015; Krupinski, Berbaum, Schartz, Caldwell, & Madsen, 2017). Specifically, detecting a lung nodule on a radiograph did not adversely affect the subsequent detection of additional lung nodules; however, it did alter observers’ willingness to report the detected nodules. The authors suggest that detecting a target during search may not induce search termination, but rather change response thresholds during a multiple-target search.

Once a diagnostician finds one target, there is no guarantee that it is the critical feature that will assist in rendering an appropriate diagnosis. It is often the case that critical features are passed over because they are not only low prevalence but also low salience; in other words, they might not stand out visually (in terms of their brightness, contrast, or geometry (Itti & Koch, 2000)) relative to background noise. Research with neurologists and pathologists has demonstrated that novice diagnosticians, such as medical residents, tend to detect features with high visual salience sooner and more often than experienced diagnosticians; this focus on highly salient visual features can be at the cost of neglecting the detection of critical features with relatively low visual salience (Brunyé et al., 2014; Matsumoto et al., 2011). In one study, not only did novice pathologists tend to fixate more on visually salient but diagnostically irrelevant regions, they also tended to re-visit those regions nearly three times as often as expert pathologists (Brunyé et al., 2014). As diagnosticians gain experience with a diverse range of medical images, features, and diagnoses, they develop more refined search strategies and richer knowledge that accurately guide visual attention toward diagnostically relevant image regions and away from irrelevant regions, as early as the initial holistic inspection of an image (Kundel et al., 2008). As described in Kundel and colleagues’ model, expert diagnosticians are likely to detect cancer on a mammogram before any visual scanning (search) takes place, referred to a an initial holistic, gestalt-like perception of a medical image (Kundel et al., 2008). This discovery led these authors to reconceptualize the expert diagnostic process as involving an initial recognition of a feature, followed by a search and diagnosis (Kundel & Nodine, 2010); this is in contrast to traditional conceptualizations suggesting that search always preceded recognition (Kundel & Nodine, 1978). Unlike experts, during the initial viewing of a medical image novices are more likely to be distracted by highly salient image features that are not necessary for diagnostic interpretation. The extent to which a medical image contains visually salient features that are irrelevant for accurate interpretation may make it more likely a novice pathologist or neurologist will be distracted by those features and possibly fail to detect critical but lower-salience image features. This might be especially the case when high-contrast histology stains or imaging techniques render diagnostically irrelevant (e.g., scar tissue) regions highly salient. Eye tracking is a critical tool for recognizing and quantifying attention toward distracting image regions and has been instrumental in identifying this source of search failure among relatively novice diagnosticians.

In a recent taxonomy of visual search errors, Cain and colleagues demonstrated that working memory resources are an important source of errors (Cain et al., 2013). Specifically, when an observer is searching for multiple features (targets), if they identify one feature they may maintain that feature in working memory while searching for another feature. This active maintenance of previously detected features may deplete working memory resources that could otherwise be used to search for lower-salience and prevalence targets. This is evidenced by high numbers of re-fixations in previously detected regions, suggesting an active “refreshing” of the contents of working memory to help maintain item memory (Cain & Mitroff, 2013). This proposal has not been examined with diagnosticians inspecting medical images, though it suggests that physicians with higher working memory capacity may show higher performance when searching for multiple features, offering an interesting avenue for future research. Together, resource depletion, low target prevalence, satisfaction of search, and distraction may account for search errors occurring across a range of disciplines involving medical image interpretation.

#### Recognition errors

Eye tracking has been instrumental in demonstrating that fewer than half of interpretive errors are attributed to failed search, suggesting that most interpretive errors arise during recognition and decision-making (Al-Moteri et al., 2017; Carmody et al., 1980; Nodine & Kundel, 1987; Samuel, Kundel, Nodine, & Toto, 1995). Recognition errors occur when the eyes fixate a feature, but the feature is not recognized correctly or not recognized as relevant or valuable for the search task. Recognition is an example of attentional mechanisms working together to dynamically guide attention toward features that may be of diagnostic relevance and mapping them to stored knowledge. One way of parsing eye movements into successful versus failed recognition of diagnostically relevant features is to assess fixation durations on critical image regions (Kundel & Nodine, 1978; Mello-Thoms et al., 2005). In this method, individual fixation durations are parsed into two categories using a quantitative threshold. For example, Kundel and Nodine used a 600-ms threshold, and Mello-Thoms and colleagues used a 1000-ms threshold; fixation durations shorter than the threshold indicated failed recognition, whereas durations lengthier than the threshold indicated successful recognition (Kundel & Nodine, 1978; Mello-Thoms et al., 2005). Thus, if a feature (e.g., a lung nodule) was fixated there was successful search, and if it was fixated for longer than the threshold there was successful recognition. Under the assumption that increased fixation durations indicate successful recognition, if a participant fixates on a particular region for longer than a given threshold then any subsequent diagnostic error must be due to failed decision-making.

Using fixation durations to identify successful recognition is an imperfect approach; it is important to note that lengthier fixation durations are also associated with difficulty disambiguating potential interpretations of a feature (Brunyé & Gardony, 2017). In other words, while previous research assumes that lengthy fixation durations indicate successful recognition, they can also indicate the perceptual uncertainty preceding incorrect recognition. This is because a strategic shift of attention toward a particular feature is evident in oculomotor processes, for instance with longer fixations, regardless of whether recognition has proceeded accurately (Heekeren, Marrett, & Ungerleider, 2008). Thus, one can only be truly certain that successful recognition has occurred (i.e., mapping a perceived feature to an accurate knowledge structure) if converging evidence is gathered during the interpretive process.

Consistent with this line of thinking, Manning and colleagues found that false-positives when examining chest radiographs were typically associated with longer cumulative dwell time than true-positives (Manning et al., 2006). Other methods such as think-aloud protocols and feature annotation may prove especially valuable to complement eye tracking in these situations: when a diagnostician recognizes a feature, they either say it aloud (e.g., “I see cell proliferation”) or annotate the feature with a text input (Pinnock, Young, Spence, & Henning, 2015). These explicit feature recognitions can then be assessed for their accuracy and predictive value toward accurate diagnosis.

In addition to measuring the ballistic movements of the eyes, eye trackers also provide continuous recordings of pupil diameter. Pupil diameter can be valuable for interpreting cognitive states and can be used to elucidate mental processes occurring during medical image interpretation. Pupil diameter is constantly changing as a function of both contextual lighting conditions and internal cognitive states. Alterations of pupil diameter reflecting cognitive state changes are thought to reflect modulation of the locus coeruleus-norepinephrine (LC-NE) system, which indexes shifts from exploration to exploitation states (Aston-Jones & Cohen, 2005; Gilzenrat, Nieuwenhuis, Jepma, & Cohen, 2010). Specifically, when the brain interprets a bottom-up signal (e.g., a salient region that attracts an initial fixation) as highly relevant to a task goal, it will send a top-down signal to selectively orient attention to that region. When that occurs, there is a transient increase in pupil diameter that is thought to reflect a shift from exploring the scene (i.e., searching) to exploiting perceived information that is relevant to the task (Privitera, Renninger, Carney, Klein, & Aguilar, 2010; Usher, Cohen, Servan-Schrieber, Rajkowski, & Aston-Jones, 1999). Recent research has demonstrated that during fixation on a scene feature, the time-course of pupil diameter changes can reveal information about an observer’s confidence in their recognition of the feature (Brunyé & Gardony, 2017). Specifically, features that are highly difficult to resolve and recognize cause a rapid pupil dilation response within a second of fixation on the feature. This opens an exciting avenue for using converging evidence, perhaps from fixation duration, pupil diameter, and think-aloud protocols, to more effectively disentangle the instances when lengthy fixations on image features are associated with successful or unsuccessful recognition. In the future, algorithms that can automatically detect instances of successful or failed recognition during fixation may prove particularly valuable for enabling computer-based feedback for trainees.

#### Decision errors

As observers gather information about a scene, including searching and recognizing features as relevant to task goals, they begin to formulate hypotheses regarding candidate diagnoses. In some cases, a hypothesis may exist prior to visual inspection of an image (Ledley & Lusted, 1959). The main function of examining a visual image and recognizing features is to develop and test diagnostic hypotheses (Sox, Blatt, Higgins, & Marton, 1988). Developing and testing hypotheses is a cyclical process that involves identifying features that allow the observer to select a set of candidate hypotheses, gathering data to test each hypothesis, and confirming or disconfirming a hypothesis. If the clinician has confirmed a hypothesis, the search may terminate; search may continue if the clinician identifies potential support for multiple hypotheses (e.g., diagnoses with overlapping features) and must continue in the service of differential diagnosis. If the clinician has disconfirmed one of several hypotheses but has not confirmed a single hypothesis, the cyclical process continues; the process also continues under conditions of uncertainty when no given hypotheses have been ruled in or out (Kassirer, Kopelman, & Wong, 1991). It is also important to keep in mind that several diagnoses fall on a spectrum with categorical delineations, with the goal of identifying the highest diagnostic category present in a given image. For instance, a breast pathologist examining histological features may categorize a case as benign, atypia, ductal (DCIS) or lobular carcinoma in situ, or invasive carcinoma (Lester & Hicks, 2016). Given that the most advanced diagnosis is the most important for prognosis and treatment, even if a less advanced hypothesis is supported (e.g., atypia), the pathologist will also spend time ruling out the more advanced diagnoses (e.g., carcinoma in situ, invasive). This may be especially the case when diagnostic features can only be perceived at high-power magnification levels, rendering the remainder of the image immediately imperceptible and making it necessary to zoom out to consider other regions.

In an ideal scenario, critical diagnostic features are detected during search and recognized, which leads the clinician to successfully develop and test hypotheses and produce an accurate diagnosis. In the real world, errors emerge at every step of that process. While decision-related errors may not be readily detected in existing eye-tracking metrics, some recent research suggests that relatively disorganized movements of the eyes over a visual image may indicate higher workload, decision uncertainty, and a higher likelihood of errors (Brunyé, Haga, Houck, & Taylor, 2017; Fabio et al., 2015). Specifically, tracking the entropy of eye movements can indicate relatively disordered search processes that do not follow a systematic pattern. In this case, entropy is conceptualized as the degree of energy dispersal of eye fixations across the screen in a relatively random pattern. Higher fixation entropy might indicate relative uncertainty in the diagnostic decision-making process. Furthermore, tonic pupil diameter increases can indicate a higher mental workload involved in a decision-making task (Mandrick, Peysakhovich, Rémy, Lepron, & Causse, 2016). No studies have examined the entropy of eye movements during medical image interpretation, and to our knowledge only one has examined pupil diameter (Mello-Thoms et al., 2005), revealing an exciting avenue for continuing research. Specifically, continuing research may find value in combining fixation entropy and pupil diameter to identify scenarios in which successful lesion detection and recognition has occurred, but the clinician is having difficulty arriving at an appropriate decision.

## Implications for medical education

Eye tracking may provide innovative opportunities for medical education, training, and competency assessment (Ashraf et al., 2018). Most existing research in this regard leverages the well-established finding that experts move their eyes differently from novices (Brunyé et al., 2014; Gegenfurtner, Lehtinen, & Säljö, 2011; Krupinski, 2005; Krupinski et al., 2006; Kundel et al., 2008; Lesgold et al., 1988). Thus, the premise is that educators can use eye tracking to demonstrate, train, and assess gaze patterns during medical education, possibly accelerating the transition from novice to expert.

Competency-based medical education (CBME) is intended to produce health professionals who consistently demonstrate expertise in both practice and certification (Aggarwal & Darzi, 2006). Though the concept of CBME has been around for several decades, formal frameworks for competency training and assessment have been more recently developed by CanMEDS, the Outcome Project of the US Accreditation Council for Graduate Medical Education (ACGME), and the Scottish Doctor (Frank & Danoff, 2007; Nasca, Philibert, Brigham, & Flynn, 2012; Simpson et al., 2002; Swing, 2007). In each of these cases, methods were evaluated and implemented for integrating CBME, including new standards for curriculum, teaching, and assessment. Many programs, however, have struggled to create meaningful, relevant, and repeatable outcome-based assessments for use in graduate medical education, residency, and fellowships (Holmboe, Edgar, & Hamstra, 2016).

### Eye tracking in medical education

As students develop proficiency in interpreting visual images, they demonstrate refined eye movements that move more quickly and consistently toward diagnostic regions of interest (Richstone et al., 2010). In other words, their eye movements increasingly resemble those of experts as they progress through training. One possible method for facilitating this progression is by showing students video-based playbacks of expert eye movements, a method called eye-movement modeling examples (EMMEs (Jarodzka et al., 2012)). Eye-movement modeling examples typically involve not only showing a video of expert eye movements, but also the expert’s audio narrative of the interpretive process (Jarodzka, Van Gog, Dorr, Scheiter, & Gerjets, 2013; van Gog, Jarodzka, Scheiter, Gerjets, & Paas, 2009). The idea that EMMEs can assist education leverages a finding from cognitive neuroscience demonstrating that observing another’s actions causes the brain to simulate making that same action (i.e., the brain’s “mirror system”), and helps students integrate the new action into their own repertoire (Calvo-Merino, Glaser, Grèzes, Passingham, & Haggard, 2005; Calvo-Merino, Grèzes, Glaser, Passingham, & Haggard, 2006). EMMEs also ground a student’s education in concrete examples, provide students with unique expert insights that might otherwise be inaccessible, and help students learn explicit strategies for processing the visual image (Jarodzka et al., 2012).

Outside of the medical domain, EMMEs have been demonstrated to help novice aircraft inspectors detect more faults during search (Sadasivan, Greenstein, Gramopadhye, & Duchowski, 2005), circuitry board inspectors detect more faults during search (Nalanagula, Greenstein, & Gramopadhye, 2006), programmers debug software faster (Stein & Brennan, 2004), students become better readers (Mason, Pluchino, & Tornatora, 2015), and novices solve puzzles faster (Velichkovsky, 1995). In medical domains involving visual image inspection, the viewed action is the sequence of an expert clinician’s fixations and saccades over the medical image, along with their verbal narration. Few studies have examined the impact of EMMEs in medical learning; note that we differentiate education from training in this context, with education involving the passive viewing of expert eye movements outside of an immediate training context (i.e., not during active practice). In the first study of this kind, novice radiographers viewed either novice or expert eye movements prior to making a diagnostic interpretation of a chest x-ray (Litchfield, Ball, Donovan, Manning, & Crawford, 2010). Viewing expert *or* novice eye movements improved a novice’s ability to locate pulmonary nodules relative to a free search, as long as the depicted eye movements showed a successful nodule search. This result suggests that novices can indeed leverage another’s eye movements to more effectively guide their own search behavior. More recently, medical students were shown case videos of infant epilepsy, in one of three conditions (Jarodzka et al., 2012). In the control condition, there was expert narration during video playback. Two experimental conditions displayed the narrated video with overlaid expert eye movements; in one condition, the eye movements were indicated by a small circle, and in the other condition, there was a “spotlight” around the circle that blurred image regions that were outside of the expert’s focus. Results demonstrated increased diagnostic performance of students after viewing the spotlight condition, suggesting that this specific condition was most effective at conveying expert visual search patterns. Thus, some research suggests that passively viewing an expert’s eye gaze can be advantageous to medical education.

While previewing an expert’s eye movements can facilitate interpretive performance on the same or very similar cases, it is unclear whether EMMEs are supporting strategy development that will transfer to dissimilar cases. Transfer describes the ability to apply knowledge, skills and abilities to novel contexts and tasks that have not been previously experienced (Bransford, Brown, & Cocking, 2000). Transfer can be relatively near-transfer versus far-transfer (Barnett & Ceci, 2002), and is considered a critical trademark of successful learning (Simon, 1983). An example of near-transfer might be a pathologist learning the features and rules for diagnosing DCIS on one case or from text-book examples, and transferring that knowledge and skill to a biopsy with similar features that clearly indicate DCIS (Roads, Xu, Robinson, & Tanaka, 2018). An example of relatively far-transfer would be successfully applying knowledge and skill to a novel biopsy with a unique cellular architecture and challenging features that are less clearly indicative of DCIS and are perhaps borderline between atypical ductal hyperplasia (ADH) and DCIS. More research is needed to understand whether EMMEs promote only near-transfer, or whether multiple EMME experiences can promote relatively far-transfer by promoting perceptual differentiation of features, accurate feature recognition, and more accurate and efficient mapping of features to candidate diagnoses. In other words, can EMMEs move beyond providing explicit hints and cues that enable interpretation and diagnosis in highly similar contexts and cases, to accelerating rule and strategy learning that enhances performance on highly dissimilar contexts and cases (Ball & Litchfield, 2017)? Second, it is worth pointing out that some research has suggested that people may intentionally alter their patterns of eye movements if they know that their eye movements are being monitored or that videos of their eye movements will be replayed to others (Neider, Chen, Dickinson, Brennan, & Zelinsky, 2010; Velichkovsky, 1995). While any such effects appear to be both rare and subtle, they do present a challenge to interpreting whether the effects of EMMEs are at least partially due to the intent of the expert viewer as opposed to being a natural representation of their viewing patterns in normal clinical practice (Ball & Litchfield, 2017).

### Eye tracking in medical training

As opposed to a novice passively viewing expert eye-gaze behavior, some studies have examined eye gaze as a training tool. As noted previously, we distinguish education from training by noting that training involves active practice of knowledge and skills, with or without feedback (Kern, Thomas, & Hughes, 1998). In most research to date, eye gaze has been used to provide immediate feedback and guidance for a novice during the active exploration of a visual stimulus. This research leverages several phenomena from the cognitive and instructional sciences. First, cueing attention toward relevant features during a training activity can promote more selective attention to cued areas and help observers remember the cued information and allocate less mental energy to the non-cued areas (De Koning, Tabbers, Rikers, & Paas, 2009). For instance, subtle visual cues, such as a momentary flash of light in a specific scene region, can selectively orient attention to that region for further inspection (Danziger, Kingstone, & Snyder, 1998). Second, watching expert eye movements can help observers recognize and learn organizational strategies for viewing and interpreting visual images, understand the expert’s intent, identify the organizational structure of the images, and better organize perceived information into mental schemas (Becchio, Sartori, Bulgheroni, & Castiello, 2008; Jarodzka et al., 2013; Lobmaier, Fischer, & Schwaninger, 2006). For instance, because experts tend to move their eyes and navigate visual images differently than novices, viewing expert eye movements and patterns of navigation behavior may help observers develop more efficient search strategies. Third, well-organized expert eye movements can help an observer recognize relations within and between images, helping them discriminate similar features and possibly promote transfer to novel cases (Kieras & Bovair, 1984). For instance, an expert may saccade intentionally between features that help the observer effectively discriminate them, possibly helping them form a more thorough understanding of how to distinguish features and associated diagnoses. It is unknown whether this refined knowledge would subsequently enable successful transfer to cases with structures and features at least partially overlapping with the learned case, suggesting an avenue for future research.

One popular way to conceptualize the utility of cueing attention toward relevant scene regions is the *Theory of Hints* (Kirsh, 2009). In this theory, when people attempt to solve problems in the real world, they rely not only upon existing knowledge (including heuristics and biases) but also the effective use of any available mental aids offered by the context. In addition to explicit verbal guidance from an instructor, or explicit feedback on worked examples, hints can also come in the form of another’s eye movements (Ball & Litchfield, 2017), which can implicitly (i.e., subconsciously) or explicitly orient attention and provide information to an observer (Thomas & Lleras, 2009a, b). As evidence for relatively implicit attention guidance, novice lung x-ray interpretation can improve when they receive implicit cueing based on an expert’s eye movements (Ball & Litchfield, 2017). In accordance with the Theory of Hints, this guidance likely provided not only a cue to orient attention toward a particular scene region, but also increased the likelihood that the area would be considered in their diagnostic interpretation. Specifically, expert cueing can help a novice calibrate the relevance and importance of a region (Litchfield et al., 2010), which can be complemented by an expert’s verbal narration. Thus, it seems that cueing an observer with expert eye movements and narration not only guides attention but can also help the student assess the expert’s intentionality and incorporate that information into their emergent interpretation. As additional evidence of this phenomenon, when expert eye gaze is superimposed during a simulated laparoscopic surgery task, novices are not only faster to locate critical diagnostic regions, but also more likely to incorporate that region into their diagnosis and ultimately reduce errors (Chetwood et al., 2012). Similarly, when novice trainees have expert eye gaze during a simulated robotic surgical task, they tended to be faster and more productive in identifying suspicious nodules (Leff et al., 2015). In both cases, cueing a trainee with expert eye movements not only gets them to fixate in a desired region, but also seems to help them understand expert intent, behave more like an expert, and develop a more accurate diagnostic interpretation.

### Eye tracking in competency assessment

In addition to cueing attention during image interpretation, eye tracking can also be used as a feedback mechanism following case interpretation. As we noted above, medical training frequently involves explicit feedback by instructors on exams and worked examples. But there are few methods for providing feedback regarding the dynamic interpretive process; for instance, how a microscope was panned and zoomed, which features were inspected, and precisely where in the process difficulties may have arisen (Bok et al., 2013; 2016; Kogan, Conforti, Bernabeo, Iobst, & Holmboe, 2011; Wald, Davis, Reis, Monroe, & Borkan, 2009). Identifying concrete metrics for use in competency assessment is critical for understanding and guiding professional development from novices to experts (Dreyfus & Dreyfus, 1986; Green et al., 2009). Indeed, a “lack of effective assessment methods and tools” is noted as a primary challenge for implementing the Milestones initiative in internal medicine education (Holmboe, Call, & Ficalora, 2016; Holmboe, Edgar, & Hamstra, 2016). The Milestones initiative is intended to provide concrete educational milestones for use in assessment of medical competencies during graduate and post-graduate medical education (Swing et al., 2013). The earliest research examining eye tracking for feedback in medicine leveraged the concept of perceptual feedback, which involves showing an observer the regions they tended to focus on during an image interpretation (Kundel, Nodine, & Krupinski, 1990). This procedure was shown to improve decision-making by providing a clinician with a second opportunity to review suspicious image regions and revise their diagnosis; this procedure might be especially advantageous given that most people do not remember where they looked during a search (Võ, Aizenman, & Wolfe, 2016).

Leveraging the concept of using one’s own eye movements as a feedback tool, one recent study suggests that eye tracking may be especially valuable for clinical feedback with emergency medicine residents (Szulewski et al., 2018). In that study, eye movements were tracked in emergency medicine residents during objective structured clinical examinations in a simulation environment. During a subsequent faculty debriefing, residents were led through an individualized debrief that included a review of their eye movements during the clinical examination, with reference to scene features focused on their associated decision-making processes. Results demonstrated that all residents deemed the inclusion of eye tracking in the debriefing as a valuable feedback tool for learning, making them more likely to actively reflect on their learning experience, constructively critique themselves and compare themselves to experts, and plan responses for future clinical scenarios (Szulewski et al., 2018). Thus, eye tracking appears to be a valuable tool for augmenting qualitative feedback of trainee performance with concrete examples and guidance to help them attend to appropriate features and incorporate them into diagnoses.

## Future research directions

As eye trackers become increasingly available to consumers, lower cost, portable, and easier to use, research on principled methods for using eye tracking for competency assessment is expected to increase (Al-Moteri et al., 2017). It is worth noting that eye trackers with high temporal and spatial resolution and coverage range (e.g., across large or multiple displays) can still be quite cost prohibitive. As eye trackers develop more widespread use, however, one can readily envision both automated and instructor-guided feedback techniques to help quantify competency and provide grounded examples for individualized feedback. In mammography, recent research demonstrates that tracking eye movements and using machine-learning techniques can predict most diagnostic errors prior to their occurrence, making it possible to automatically provide cueing or feedback to trainees during image inspection (Voisin et al., 2013). In diagnostic pathology, automated feedback may be possible by parsing medical images into diagnostically relevant versus irrelevant regions of interest (ROIs) using expert annotations and/or automated machine-vision techniques (Brunyé et al., 2014; Mercan et al., 2016; Nagarkar et al., 2016). Once these ROIs are established and known to the eye-tracking system, fixations can be parsed as falling within or outside of ROIs. This method could be used to understand the spatial allocation of attention over a digital image (e.g., a radiograph, histology slide, angiography), and the time-course of that allocation.

While eye tracking provides valuable insights into the distribution of visual attention over a scene, it is important to realize that eye trackers are restricted to monitoring foveal vision. The fovea is a small region in the center of the retina that processes light from the center of the visual field, with a dense concentration of cone receptors that provide high visual acuity (Holmqvist et al., 2011). One popular theoretical assumption is that eye and head movements strategically position the retina to a more advantageous state for gathering information, such as moving your head and eyes toward the source of a sound to reveal its nature and relevance (Xu-Wilson, Zee, & Shadmehr, 2009). Thus, some of what we consider overt visual attention should theoretically be captured by tracking eye movements. On the other hand, it is also well-established that visual attention can be shifted and sustained covertly, allowing one to fixate the eyes on an ostensibly uninteresting or irrelevant feature while covertly attending to another (Liversedge & Findlay, 2000; Treisman & Gelade, 1980). Thus, it remains possible that some of a diagnostician’s interpretive process may occur through peripheral vision (parafoveal vision), limiting our interpretation of eye-tracking patterns made during medical image inspection.

Eye trackers are designed to track eye gaze as a series of fixations and saccades; in other words, they are designed to track foveal attention. This means that they are quite good at tracking overt central visual attention, but they are not intended for tracking covert peripheral visual attention (Holmqvist et al., 2011). However, we also know that visual attention can be covertly shifted to other areas of a visual scene without a subsequent overt fixation on that region (Liversedge & Findlay, 2000; Treisman & Gelade, 1980). This is typically considered a major downfall of eye tracking: that many real-world visual tasks likely involve both covert *and* overt visual attention, though eye tracking can only measure the latter. However, more recent research has demonstrated that microsaccades reflect shifts in covert attention (Meyberg, Werkle-Bergner, Sommer, & Dimigen, 2015; Yuval-Greenberg, Merriam, & Heeger, 2014). Microsaccades are very small saccades that are less than 1° of visual arc and occur very frequently during fixations (about two to three times per second) (Martinez-Conde, Otero-Millan, & MacKnik, 2013). These microsaccades tend to be directional, for instance moving slightly to the left or right of a current fixation point; research has recently demonstrated that these slight directional movements of the eye indicate the orientation of covert attention (Yuval-Greenberg et al., 2014). For example, if you are staring at a point on a screen but monitoring an upper-right area of the periphery for a change, then microsaccades are likely to show a directional shift toward the upper right. Microsaccades are likely to serve many purposes, such as preparing the eye for a subsequent saccade to a peripheral region (Juan, Shorter-Jacobi, & Schall, 2004), but can also provide meaningful metrics of covert attention. With a clinician, it is possible that while they fixated on a given number of regions they also considered additional image regions for fixation (but never visited them). In other words, microsaccades may provide more fine-grained understanding of the strategic search process within individual fixations and allow a more nuanced understanding of which regions might have been ruled-out or ruled-in for subsequent inspection.

Eye tracking also carries value for understanding longitudinal aspects of competency progression in medical education. While diagnostic performance is routinely evaluated through credentialing and certification, we have very little insight into the underlying interpretive process or the process of skills development over time. For instance, within the domain of diagnostic pathology, we know of only one study that examined longitudinal changes in pathology residents’ visual expertise (Krupinski et al., 2013). Unfortunately, this prior study is limited by its size and breadth (four residents at a single training location), the restriction of observers’ ability to zoom or pan the medical image, and a reliance on the same experimental images each year. Thus, most of our understanding of how image interpretation and diagnostic accuracy and efficiency emerge during professional development is restricted to insights from cross-sectional designs. But we also know that expertise development of medical students and post-graduate resident trainees is a long-term, continuous, and non-linear process. Eye tracking provides an innovative opportunity to enable a large-scale examination of how interpretive and diagnostic skills develop through multi-year residencies and into professional practice. Our current research is examining this exciting possibility.

We have focused primarily on competency development through education and training, and performance differences between novices and experts. However, it is worth pointing out that each individual student and clinician brings a unique set of individual differences to clinical diagnostics that undoubtedly influences the processes of visual search and decision-making. Individual differences include variables such as personality traits and cognitive abilities, and a substantial body of research demonstrates that these variables constantly influence real-world behavior (Motowildo, Borman, & Schmit, 1997). For instance, recent research has demonstrated that experienced radiologists show superior perceptual abilities to novices, as measured with the Vanderbilt Chest Radiograph Test (Sunday, Donnelly, & Gauthier, 2017). Here we consider one individual difference that warrants more consideration in the domains of medical image interpretation and decision-making: working-memory capacity. Generally, working memory refers to the cognitive system involved in maintaining and manipulating task-relevant information while a task is performed (Miyake & Shah, 1999). Working-memory capacity describes the notion that working memory is a limited capacity system: it has finite resources for processing and storage, and each person has a different resource pool that can be drawn from to successfully perform a task (Kane & Engle, 2002, 2003). To measure working memory capacity, one popular task (the operation span task) involves participants solving arithmetic problems while also trying to memorize words (Turner & Engle, 1989). In this manner, the task demands working-memory storage (to memorize the words) while also processing distracting arithmetic problems. The ability to maintain performance on a task in the face of distraction is a hallmark characteristic of individuals with high working-memory capacity. In our discussion of search errors, we noted that working memory may be critical for helping an observer maintain previously viewed features in memory while exploring the remainder of an image and associating subsequently identified features with features stored in working memory (Cain et al., 2013; Cain & Mitroff, 2013). In this case, higher working-memory capacity may be particularly important when there are multiple targets (rather than a single target) to be identified in an image. Furthermore, in our discussion of decision errors, we noted that some theories suggest that candidate hypotheses must be maintained in memory while evidence is accumulated during image inspection (Patel et al., 2005; Patel & Groen, 1986; Patel, Kaufman, & Arocha, 2002). Other theories suggest that hypotheses are formed early on and then tested during image inspection (Ledley & Lusted, 1959); it is important to point out that novices and experts may reason very differently during case interpretation, and one or both of these approaches may prove appropriate for different observers. Some research demonstrates that individual differences in working memory capacity predict hypothesis generation and verification processes in a task involving customer order predictions (Dougherty & Hunter, 2003). Thus, in both search and decision-making there appear to be critical roles for working-memory capacity in predicting clinician performance. This possibility has not yet been examined in the context of medical image interpretation and diagnosis, and it is unclear how working-memory capacity might influence clinician eye movements, though it is an exciting direction for future research.

In our review of the literature, we also noted that most studies using eye tracking during medical image interpretation use static images. These include lung x-rays, histology slides, and skin lesions. This is not entirely surprising, as many medical images are indeed static, and interpreting eye movements over dynamic scenes can be very complex and time-consuming (Jacob & Karn, 2003; Jarodzka, Scheiter, Gerjets, & van Gog, 2010). There are also cases where images that are usually navigated (panned, zoomed) are artificially restricted, increasing the risk that results are no longer relevant to routine clinical practice. As modern technologies emerge in diagnostic medicine, this disconnect becomes increasingly disadvantageous. Indeed, many medical images are becoming more complex and dynamic; for example, interpreting live and replayed coronary angiograms, simulated dynamic patients during training, or navigating multiple layers of volumetric chest x-rays (Drew, Võ, & Wolfe, 2013; Rubin, 2015). Continued innovations in software for integrating dynamic visual scenes and eye movements will enable this type of research: for instance techniques that parse dynamic video stimuli based on navigation behavior (pause, rewind, play) to identify critical video frames (Yu, Ma, Nahrstedt, & Zhang, 2003). Some other techniques are being developed to provide rudimentary tagging and tracking of identifiable objects in a scene (Steciuk & Zwierno, 2015); such a technique might prove valuable for tracking a region of diagnostic interest that moves across a scene during playback (e.g., during coronary angiogram review).

It is also worth pointing out that many hospitals are introducing mandatory consultative expert second opinions for quality assurance purposes. For instance, Johns Hopkins Hospital and the University of Iowa Hospitals and Clinics introduced mandatory second opinions for surgical pathology (Kronz, Westra, & Epstein, 1999; Manion, Cohen, & Weydert, 2008). Not only are these mandates seen as valuable for the institutions involved (e.g., for reducing malpractice suits), but clinicians also perceive them as important for improving diagnostic accuracy (Geller et al., 2014). However, having an earlier physician’s interpretation available during diagnosis may unintentionally bias the second physician’s diagnostic process. Indeed even a subtle probabilistic cue (e.g., a red dot that suggests an upcoming image contains a blast cell) can produce response bias in experienced diagnosticians (Trueblood et al., 2018). Thus, while viewing an expert’s behavior may prove advantageous in certain conditions, future research must isolate the parameters that may dictate its success and balance the potential trade-off between guiding eye movements and potentially biasing interpretation. Furthermore, second opinions can also induce diagnostic disagreements among expert clinicians and necessitate time and expense for resolving disagreement and reaching a consensus diagnosis. Eye tracking may prove to be an invaluable arbiter for these sorts of disputes, allowing consultative physicians to view the eye movements of the physician who rendered the primary diagnosis. This practice may assist in helping the consultative physician understand which features were focused on, which features were missed, and understanding how the original physician arrived at their interpretation. Eye tracking could thus augment traditional text annotations to allow consultative physicians to see the case “through the eyes” of the other physician, possibly reducing disagreement or facilitating consensus through shared understanding. Similar strategies might be applied to peer cohorts or medical students and residents, allowing them to learn from each other’s search patterns and successes and failures. On the other hand, this approach could introduce bias in the second physician and unintentionally increase agreement; if the first physician arrived at an incorrect interpretation, such agreement could be detrimental, demonstrating the importance of continuing research in this regard (Gandomkar, Tay, Brennan, Kozuch, & Mello-Thoms, 2018).

## Conclusion

Medical image interpretation is a highly complex skill that influences not only diagnostic interpretations but also patient quality of life and survivability. Eye tracking is an innovative tool that is becoming increasingly commonplace in medical research and holds the potential to revolutionize trainee and clinician experiences.

## Abbreviations

ADH: Atypical ductal hyperplasia
CBME: Competency-based medical education
DCIS: Ductal carcinoma in situ
EMME: Eye-movement modeling examples
LC-NE: Locus coeruleus-norepinephrine
ROI: Region of interest
SMI REDm: SensoMotoric Instruments’ Remote Eye-tracking Device – mobile
VNPI: Van Nuys Prognostic Indicator
